# Hepatobiliary organoid research: the progress and applications

**DOI:** 10.3389/fphar.2025.1473863

**Published:** 2025-02-11

**Authors:** Rui-Qi Zou, Yu-Shi Dai, Fei Liu, Si-Qi Yang, Hai-Jie Hu, Fu-Yu Li

**Affiliations:** ^1^ Division of Biliary Surgery, Department of General Surgery, West China Hospital, Sichuan University, Chengdu, Sichuan, China; ^2^ Research Center for Biliary Diseases, West China Hospital, Sichuan University, Chengdu, Sichuan, China

**Keywords:** organoid, stem cells, hepatobiliary disease, disease modeling, personalized medicine (PM)

## Abstract

Organoid culture has emerged as a forefront technology in the life sciences field. As “*in vitro* micro-organs”, organoids can faithfully recapitulate the organogenesis process, and conserve the key structure, physiological function and pathological state of the original tissue or organ. Consequently, it is widely used in basic and clinical studies, becoming important preclinical models for studying diseases and developing therapies. Here, we introduced the definition and advantages of organoids and described the development and advances in hepatobiliary organoids research. We focus on applying hepatobiliary organoids in benign and malignant diseases of the liver and biliary tract, drug research, and regenerative medicine to provide valuable reference information for the application of hepatobiliary organoids. Despite advances in research and treatment, hepatobiliary diseases including carcinoma, viral hepatitis, fatty liver and bile duct defects have still been conundrums of the hepatobiliary field. It is necessary and crucial to study disease mechanisms, establish efficient and accurate research models and find effective treatment strategies. The organoid culture technology shed new light on solving these issues. However, the technology is not yet mature, and many hurdles still exist that need to be overcome. The combination with new technologies such as CRISPR-HOT, organ-on-a-chip may inject new vitality into future development.

## 1 Introduction

### 1.1 Definition of organoids

**FIGURE 1:**
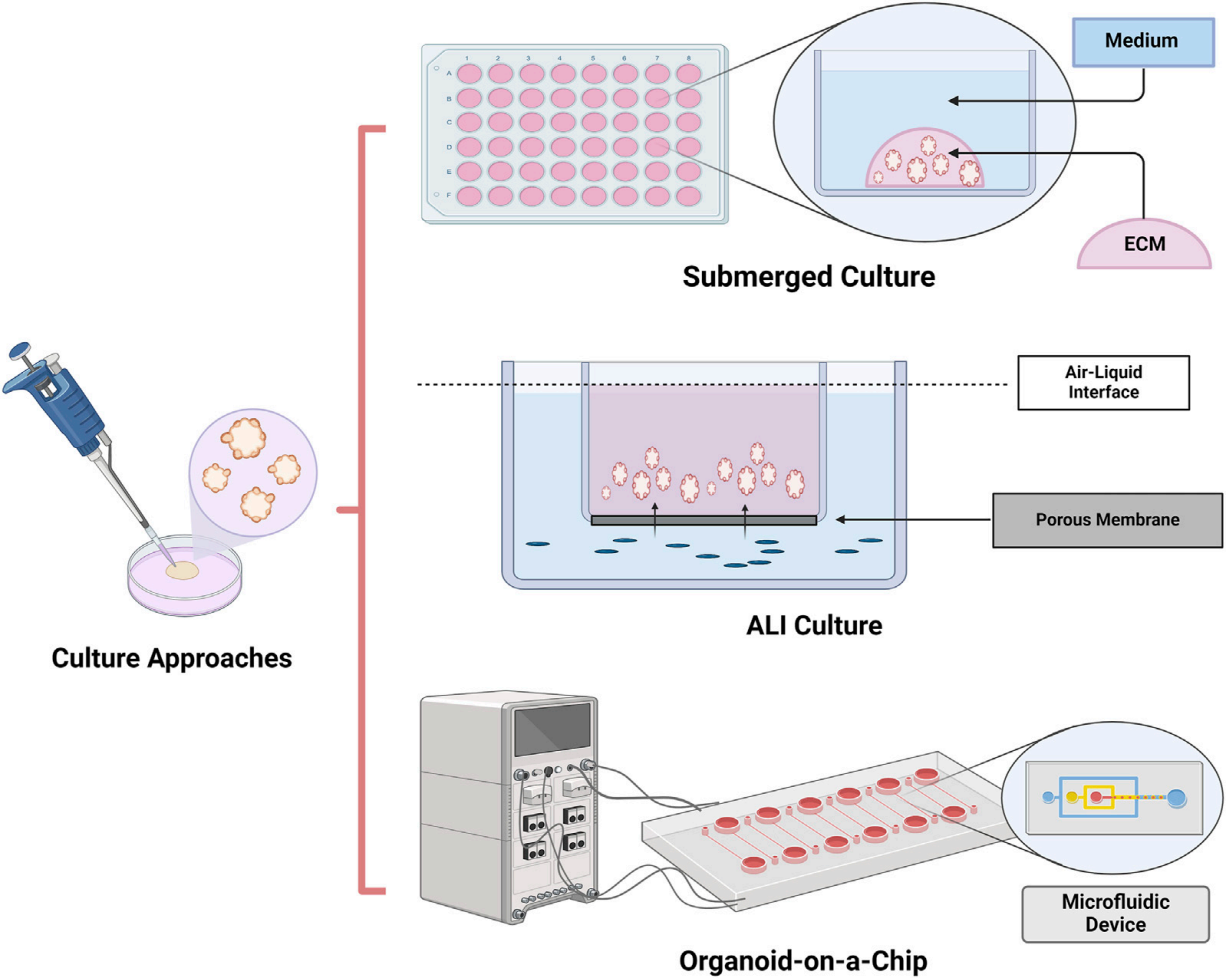
Culture approaches of organoids. Organoids can be achieved by submerged culture, air–liquid interface (ALI) culture and Organoids-on-a-chip culture. Submerged culture is the most widely used organoids culture method. Because of direct exposure to oxygen, ALI cultures provide higher oxygen supplement than submerged culture. Organ-on-a-chip is a microfluidic cell culture device which could accurately control the abiochemical and biophysical environment for cell growth.

Organoids are described as intricate 3-dimensional (3D) structures originating from human (pluripotent) stem cells, progenitor, and/or differentiated cells. They have the ability to self-assemble and differentiate into functional clusters of multiple cells, accurately reproducing the function, organization, and genetic characteristics of the original organs *in vivo* (Dutta et al., 2017; Marsee et al., 2021). Traditional 2-dimensional (2D) cell culture technique is used the most and has the advantages of convenience and simplicity. However, 2D attachment leads to cells losing their morphology and influences the organization of the structures inside the cell, proliferation, growth and differentiation, secretion, signal transduction and drug response (Baker and Chen, 2012; Scalise et al., 2021), with heterogeneity gradually obliterated, genomics and metabolomics significantly dissimilated during long-term subculture (Bresnahan et al., 2020). The organoid technology, as a 3D culture system, is created through suspension culture to prevent direct physical contact with the plastic dish, in contrast with the 2D culture method. The establishment of the 3D environment mainly relies on biological or synthetic scaffolds similar to the extracellular matrix. Furthermore, scaffold-free methods (Dituri et al., 2021), “air-liquid-interface” methods (Scalise et al., 2021; Neal et al., 2018; Lamers et al., 2021; Wakamatsu et al., 2022) and “Organ-on-a-chip” (Xue et al., 2021; Huang et al., 2021; Xie et al., 2022) can also be utilized to attain the 3D structure of the organoids (Figure 1). The intricate surrounding milieu regulates the structure, development and function of cells in the organism, encompassing interactions between cells and cell-extracellular matrix (ECM). Because Matrigel plays an excellent supporting role, the 3D culture conditions can recapitulate the microenvironment in which primary cells are located accurately (Tuveson and Clevers, 2019). In this scenario, 3D-grown organoids exhibit strong resemblance to the parents, and also retain the genetic stability and chromatin heterogeneity of the parents. Additionally, organoids can proliferate quickly within 1–2 weeks and can be stably sub-cultured and cryopreserved similar to normal cell lines (Drost and Clevers, 2018). In addition, cells are able to aggregate into spherical shapes under 3D culture conditions, which contributes to establishing the intercellular signaling pathways (Fan et al., 2019). Organoid models have characteristics similar to living organs: 1) they contain various organ-specific cell types; 2) exhibiting some specific functions related to organs; 3) forming a spatial structure similar to organs. Organoid are considered as an important model in exploring the occurrence, progression and evolution of diseases due the ability of faithfully replicating and simulating the distinctive biological traits of organs and parent cells. Moreover, tumor organoids can be established through preoperative biopsy or postoperative resection specimen, serving a crucial function in predicting personalized drug sensitivity and screening adjuvant therapy medications. Therefore, organoid models offer superior alternatives for drug screening and personalized drug treatment (Broutier et al., 2017; Wang et al., 2021; Yuan et al., 2022). The recognition of organoids’ potential to broaden fundamental research by supplementing existing model systems is becoming more widespread (Bahmad et al., 2021).

**FIGURE 2:**
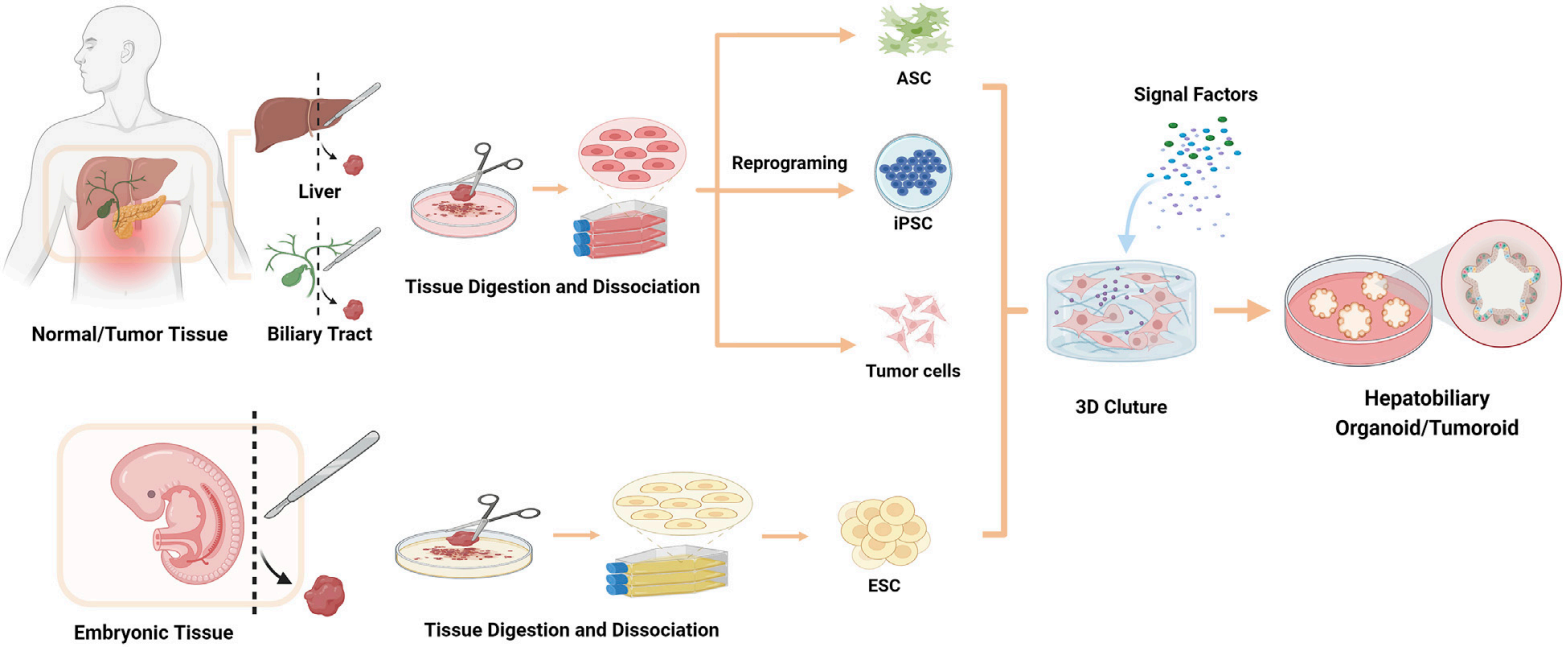
Strategies for organoids/tumoroids establishment *in vitro*. The cell sources for establishing organoids include ESCs, ASCs, iPSCs and tumor cells.

Stem cells, being primitive and undifferentiated, possess the capability to differentiate into distinct and specialized cell categories. Organoids can be derived from embryonic stem cells (ESCs), induced pluripotent stem cells (iPSCs), and adult stem cells (ASCs), due to the self-renewal and multi-directional differentiation potential of stem cells (Figure 2) (Lancaster and Knoblich, 2014; Takebe and Wells, 2019). The development of organoids from stem cells is comparable to how the organ obtains its unique organizations, primarily involves the self-organization of the cell population (Rossi et al., 2018). It needs to mimic an *in vivo* microenvironment and active various signaling pathways during cell development and differentiation to induce self-organization. Organoids derived from pluripotent stem cells (PSCs, including ESCs or iPSCs) are established through directed differentiation of PSCs. To initiate cell-directed differentiation and maturation, it is necessary to form particular germ layers (endoderm, mesoderm or ectoderm) and then co-culture them with specific growth and signaling factors as well as cytokines. Culturing of ASCs-derived organoid require to isolate the tissue-specific stem cells from the target organ, and then embed them into an ECM containing defined, tissue-specific combinations of growth factors to support propagation (Huch and Koo, 2015; Kim et al., 2020). While initial studies suggested organoids were solely derived from stem cells (Lancaster and Knoblich, 2014), it is now evident that organoids can also originate from differentiated cells like cholangiocytes (Aloia et al., 2019; Sampaziotis et al., 2017).

Organoids are categorized into distinct groups based on defining characteristics, according to the consensus on the definition and nomenclature of hepatic, pancreatic and biliary organoids. These encompass epithelial, multi-tissue, and multi-organ ones. Epithelial organoids represent the most widely studied organoid type. These structures originate from one germ layer (endoderm, mesoderm, or ectoderm) and can self-renew under suitable culture conditions. Multi-tissue organoids are formed by co-culturing cells from two or more germ layers or by co-differentiating PSCs. Multi-organ organoids represent the most intricate and least documented category of organoids, characterized by inter-organ developmental self-organization patterns. These systems offer significant potential for researching organogenesis, a process regulated by various boundary tissue interactions (Marsee et al., 2021).

### 1.2 The current status

After Clevers’ team in the Netherlands published their findings in 2009, reporting that leucine-rich repeat-containing G protein-coupled receptor 5 (Lgr5) positive ASCs in the mouse intestine were capable of forming the crypt-villus structure *in vivo*, organoid models of normal organs and tumor tissues can be observed in studies of multiple fields, including the stomach, colon, pancreas, kidney, prostate, brain, and retina (Barker et al., 2010; Spence et al., 2011; Sato et al., 2011; Gao et al., 2014; Eiraku and Sasai, 2011; Lancaster et al., 2013; Low et al., 2019; Boj et al., 2015).

In 2013, the 3D culture method was used by Takebe’s lab in the construction of 3D vascular and functional iPSC-derived liver buds (iPSC-LBs) *in vitro*. The analysis of immunostaining and gene expression demonstrated the resemblance between liver buds induced *in vitro* and those *in vivo*. Moreover, the internal functional blood vessels could promote the maturation of iPSC-LBs into liver tissue (Takebe et al., 2013). Takebe’s study addressed the technical challenges of organoid boundary system formation, opening up the possibility of studying complex interactions during early organ development.

In the same year, Clevers et al. extracted Lgr5^+^ progenitor-like oval cells from the portal triad area in injured mouse livers that CCl_4_ induced. Then, Lgr5^+^ cells were cultured and induced to differentiate in a Matrigel matrix with Wnt3a, R-pondin-1, EGF, HGF, FGF10 and Noggin to establish mature mouse hepatobiliary organoids (Huch et al., 2013). Certain liver progenitor cells could develop into early hepatocytes and biliary epithelial cells, according to the analysis of the resulting organoids. Which demonstrated that the mouse liver organoids were bipotential. Under the culture conditions that added Notch, TGF-β pathway inhibitors, FGF, BMP7, EGF, and dexamethasone without R-spondin-1 and HGF, these bipotential organoids tended to differentiate into hepatocytes. After implantation into immunodeficient mice, mouse liver organoids differentiated into liver tissues and showed mature hepatocyte markers and function, including low-density lipoprotein uptake, albumin and bile acid secretion, glycogen accumulation, and induction of the cytochrome P450 system (Huch et al., 2013; Schulze et al., 2019). Soon after in 2015, Clevers et al. successfully established human liver organoids originated from EpCAM^+^ cells obtained from the human liver *in vitro*, which were bipotential (Huch et al., 2015). In 2018, studies showed that more mature and longer-lasting hepatocyte organoids could be constructed by inducing purified AXIN2^+^ mouse hepatocytes (Peng et al., 2018; Hu et al., 2018). Furthermore, there have been reports of hepatocyte organoids originated from human embryonic liver tissue of aborted fetuses (Hu et al., 2018). Wang et al. established human ESCs derived expandable hepatic organoids (hEHOs) from using a new type of media (serum-free, feeder-free). The hEHOs were capable of maintaining the phenotypic traits of bipotential hepatic stem cells stably and had the ability to differentiate into functional hepatocytes or cholangiocytes (Wang et al., 2019). Wu et al. successfully established the first functional hepatobiliary organoids (HBOs) using human induced pluripotent stem cells (hiPSCs) (Wu et al., 2019). The authors produced hepatobiliary organoids by inducing hiPSCs to form endoderm and mesoderm tissues simultaneously and activating the NOTCH2 and TGF-β signaling pathways to generate separate hepatocyte and cholangiocyte populations. Next, the hepatobiliary organoids were matured using a proprietary cholesterol^+^ MIX supplemented standard base medium. Soon after, Wu et al. updated their previous protocol (Wu et al., 2019), which shortened the time to achieve maturation *in vitro*, and developed a medium that could maintain HBOs for more than 1.5 months (Wu et al., 2021). Takebe’s team successfully constructed the continuous and dynamic hepato-biliary-pancreatic organoid (HBPO). Furthermore, a functional connection between the internal pancreas, especially the exocrine lineage, and the bile ducts within HBPO (Koike et al., 2019). Functioning human liver organoids were generated from pluripotent stem cells derived from peripheral blood CD34^+^ cells by Kasem et al. (Kulkeaw et al., 2020). Since only the hepatic endoderm was able to form liver organoids without co-culture with the endothelium and septum mesenchyme, endothelial cells or hepatic progenitor cells (Takebe et al., 2013; Pettinato et al., 2019; Ng et al., 2018), the method of Kasem et al. was simple and faster than a previous study (Mun et al., 2019). This study also showed that hiPSCs produced from hematopoietic progenitor cells could differentiate into hepatocytes and create liver organoids, indicating that a less invasive approach could be used to manufacture hiPSCs. Wendy et al. constructed multi-cellular human liver PSC-derived organoids, comprised predominantly hepatic epithelial cells, differentiated simultaneously with stellate-like and hepatic macrophage-like cell that had the potential for modeling of hepatic inflammatory diseases *in vivo* (Thompson and Takebe, 2020).

Research have shown that self-renewing epithelial organoids can be cultured from primary tissue of the human liver (Huch et al., 2015; Hu et al., 2018) and extrahepatic biliary tree (Sampaziotis et al., 2017; Lugli et al., 2016). Self-organizing 3D structures could also be cultured from primary and metastatic tumors and even tumor needle biopsies of the liver and extrahepatic bile ducts (Broutier et al., 2017; Nuciforo et al., 2018; Saito et al., 2019). Hepatocellular carcinoma (HCC)-derived organoids replicate the histological structure, mutation profile, and transcriptome of the original tumor. The same applied to the culture of intrahepatic cholangiocarcinoma organoids, which maintained their drug-resistance phenotype, enabling in-depth mechanistic and personalized drug interaction research.

## 2 Applications of hepatobiliary organoids

**FIGURE 3:**
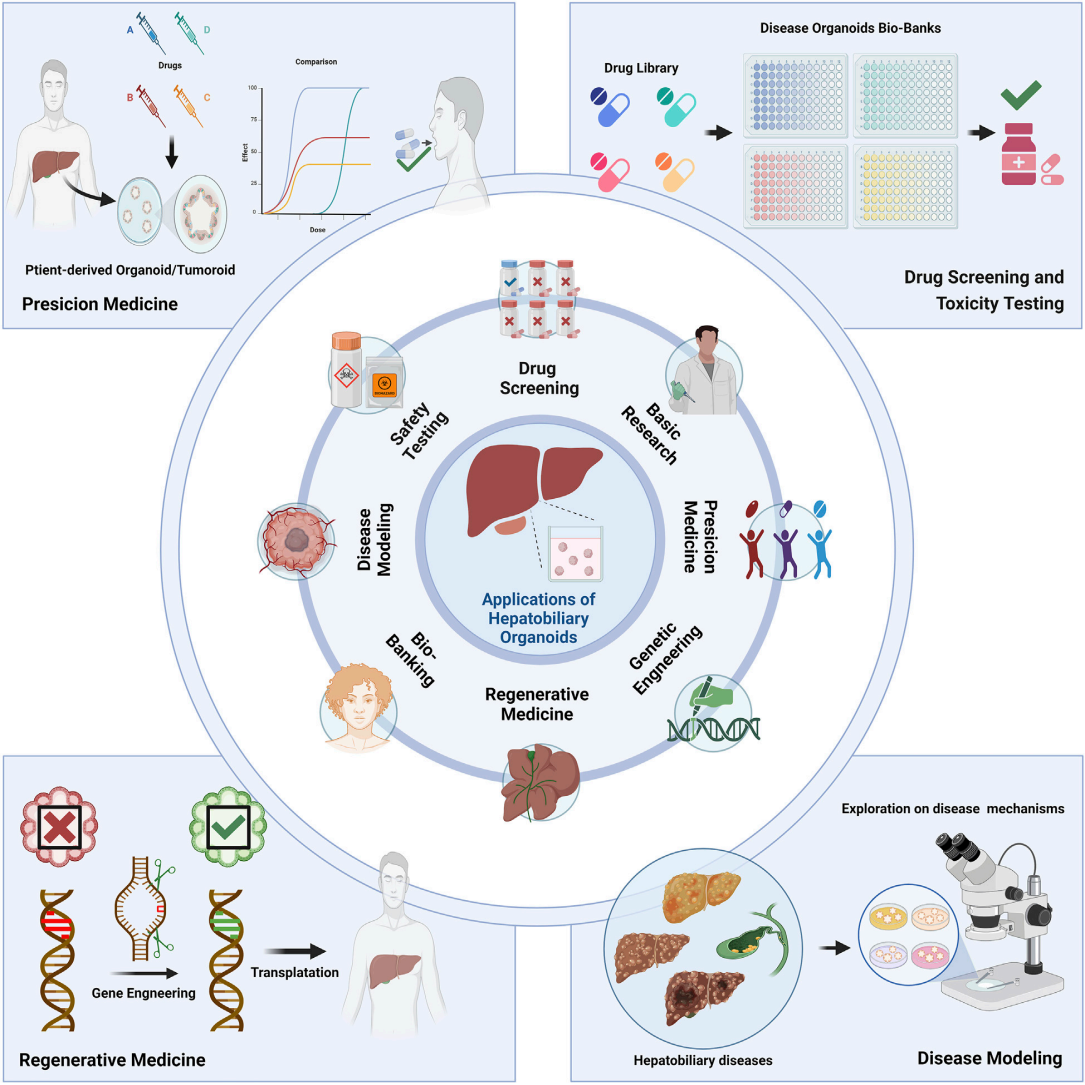
Applications of the hepatobiliary organoids. Organoids have wide application prospects on basic research, drug screening, safety testing, disease modeling, bio-banking, regenerative medicine, genetic engineering, precision medicine and many other fields.

Organoid technology has significant advantages: 1) Human-derived: Human organoids represent human physiology, 2) Rapid: Organoids can be rapidly and easily established derived from ASC and PSC, 3) Robustness: Scale-up is usually possible for drug and genomic screening on a large scale, once established, 4) Genetic manipulation: majority of genetic engineering tools can be used on iPSC or directly on organoid systems, 5) Personalization: iPSCs and organoids can be obtained from individuals (Kim et al., 2020). The wide array of biomedical applications (Figure 3) is facilitated by these benefits of hepatobiliary organoids.

### 2.1 Hepatobiliary disease research

#### 2.1.1 Hereditary disease

Organoids are able to be used to study and model organ-specific genetic diseases. Alpha-1 antitrypsin (AAT) deficiency (AATD), one of the inherited metabolic diseases, results from deficiency of the anti-protease component-α1-antitrypsin in the blood. Clevers et al. used biopsies from patients with α1-antitrypsin deficiency to generate liver organoids by organoid culture technology (Huch et al., 2015). They observed AAT protein aggregates in the resulting organoids, which resembled the findings in the original biopsy. Besides, supernatants from these organoids showed a lower ability to block elastase activity. In addition, a Spanish team’ success confirming Clevers’s findings (Gómez-Mariano et al., 2020).

Alagille syndrome (ALGS) is a rare multisystem disorder caused by mutations of the JAG-1 and NOTCH2 genes (ShenTu et al., 2021; Mitchell et al., 2018). The main hepatobiliary presentations are biliary atresia and chronic cholestasis caused by bile duct hypoplasia. The team of Clevers reported that they had established the first human ALGS liver organoid models (Huch et al., 2015). When R-spondin, Nicotinamide, TGFbi and FSK were removed, the ALGS liver organoids lost the potential to upregulate biliary markers. A similar conclusion was drawn from the study of Emma et al. (Andersson et al., 2018). Guan et al. introduced the mutation causing ALGS in JAG1 with Clustered regularly interspaced short palindromic repeat (CRISPR)/CRISPR-associated protein 9 (Cas9) technology and cultured and induced iPSCs from healthy people to produce liver organoids whose pathological features were similar to ALGS (Guan et al., 2017).

Polycystic liver disease, also known as cystic fibrosis (CF), caused by mutations in a cell-surface chloride transporter called cystic fibrosis transmembrane regulator (CFTR) gene (Masyuk et al., 2022; Kothadia et al., 2024). Sampaziotis’ team applied 3D culture technology to construct cystic fibrosis organoid models with cholangiocytes generated by inducing PSCs (Sampaziotis et al., 2015). Monique et al. established cholangiocyte organoids by extrahepatic cholangiocytes obtained from a compound CFTR gene mutation patient (Verstegen et al., 2020).

Wilson’s disease, also known as hepatolenticular degeneration, is an autosomal recessive copper metabolism disorder, manifest as accumulation of copper ions in major organs such as the liver (Lucena-Valera et al., 2021; Arai et al., 2021). Nantasanti et al. segregated hepatocytes from copper metabolism MURR1 domain 1 (COMMD1) deficient dogs to culture organoids and observed the intracellular copper accumulation, which demonstrated that the *in vitro* model of Wilson’s disease was generated successfully (Nantasanti et al., 2015). In 2020, a study isolated and cultured hepatic progenitors from COMMD1-deficient dogs to generate organoids (Kruitwagen et al., 2020). After gene correction, the organoid-derived hepatocyte-like cells are transplanted via the portal vein into the dog livers, and the cells engraft and survive up to 2 years. This study provided a new approach that applied organoids as tools to treat gene-defective inherited liver diseases.

Wolman disease (WD) is an autosomal recessive genetic disease caused by the inactivation of acid lipase in the lysosome (Aguisanda et al., 2017). A large amount of lipid accumulates in the hepatocytes contribute to steatohepatitis and fibrosis (Aguisanda et al., 2017; Pastores and Hughes, 2020). To explore new treatment methods, Ouchi et al. constructed three organoid models with severe fibrosis by culturing and inducing iPSCs of Wolman disease patients (Ouchi et al., 2019). They added FGF19, which could relieve symptoms of WD into the culture system of organoids and found that the production of reactive oxygen species, a marker of hepatocyte injury in nonalcoholic fatty liver, was significantly decreased (Attia et al., 2017).

#### 2.1.2 Viral hepatitis

Viral hepatitis, especially hepatitis B and hepatitis C, is one of the major public health problems and economic burdens worldwide (Fotiadu et al., 2004; Shiha et al., 2020; Zhang et al., 2021). Two studies had used pluripotent stem cells from hepatitis B patients and liver specimens from hepatitis C patients to induce and culture liver organoids with hepatitis B and hepatitis C, respectively (Nie et al., 2018; Baktash et al., 2018). According to recent studies, liver organoids obtained from healthy individuals were co-cultured with either the recombinant virus or the serum of patients with HBV. As a result, the organoids became infected and the virus showed active proliferation (De Crignis et al., 2021). The 3D organoid models of viral hepatitis can demonstrate the connection and interaction between the hepatitis virus and host cells, offering essential preclinical models for mechanism research, drug discovery and treatment of viral hepatitis.

#### 2.1.3 Fatty liver

Recently, organoids have been recognized as the favored 3D *in vitro* representation for studying non-alcoholic fatty liver disease (NAFLD) (Ramos et al., 2022). Ouchi’ team, using PSC lines, developed a reproducible method to derive multi-cellular human liver organoids composed of hepatocyte-, stellate-, and Kupffer-like cells. Under treatment of free fatty acid, organoids recapitulated steatosis, inflammation and fibrosis phenotypes, which are key characteristics of steatohepatitis, successively. Gurevich et al. established a novel *in vitro* differentiation process to generate cryopreservable hepatocytes using an iPSC panel of non-alcoholic steatohepatitis (NASH) donors and healthy controls (Gurevich et al., 2020). In drug metabolism research field, team of McCarron developed methods that allow the derivation, proliferation, hepatic differentiation, and extensive characterization of bipotent ductal organoids from NASH patients’ irreversibly damaged live (McCarron et al., 2021). Hendriks’ team introduces the FatTracer, a CRISPR screening platform designed to identify steatosis modulators and potential targets using APOB^−/−^ and MTTP^−/−^ organoids and identified fatty acid desaturase 2 (FADS2) as a key factor in hepatic steatosis. These organoid models enable the investigation of steatosis causes and drug targets (Hendriks et al., 2023). Kimura et al. devised a pooled human organoid-panel of steatohepatitis to investigate the impact of metabolic status on genotype-phenotype association. ‘‘In-a-dish’’ genotype-phenotype association strategies disentangle the opposing roles of metabolic-associated gene variant functions and offer a rich mechanistic, diagnostic, and therapeutic inference toolbox toward precision hepatology (Kimura et al., 2022). Consequently, organoid-derived fatty liver models are central tools to further study the occurrence, transformation and mechanism of steatosis disease.

#### 2.1.4 Biliary atresia and bile duct defects

Biliary atresia (BA) is characterized by progressive extrahepatic and intrahepatic biliary fibrosis and biliary obstruction. If left untreated, affected patients will eventually develop portal hypertension and liver failure (Lin et al., 2019; Vij and Rela, 2020). BA mainly occurs in neonates, and the etiology is still unclear, which may be related to viral infection, immune damage, environmental and genetic factors (Vij and Rela, 2020). BA is the main indication for pediatric liver transplantation (Zhou et al., 2019). After infection of human cholangiocyte organoids with rotavirus, severe cytopathic changes occurred in the organoid cells, which could partially mimic the development and pathological changes of BA (Chen et al., 2020). Sinobol et al. treated mouse liver ductal organoids with acetaminophen and found that the expression of fibrogenic cytokines and cholangiocyte apoptosis increased, indicating that the organoid model can simulate injury-induced apoptosis of cholangiocytes in BA (Chusilp et al., 2020). Bile duct epithelium organoids were cultured with biliatresone, the cell structure of organoids was destroyed, and the normal apical-basal structure was lost (Lorent et al., 2015). The phenomenon of breakdown in apical-basal polarity was also observed in organoids derived from BA patients or rhesus rotavirus A-infected mice (Babu et al., 2020), which has been confirmed in a recent study (Amarachintha et al., 2022). Cholangiocyte organoids derived from liver biopsies of BA patients showed low expression of developmental and functional markers (cytokeratin 7, EpCAM, transporters aquaporin 1, CFTR), small quantity and misorientation of cilia, a change in the expression pattern of zonula occludens-1 (ZO-1) and increased permeability (Amarachintha et al., 2022). The results above proved that BA patient-derived organoids are excellent models for studying the deficiency of molecular and function in the delayed development of cholangiocytes in BA.

Iatrogenic bile duct injury has become one of the most common causes of benign bile duct defects or strictures with the popularization and broad application of laparoscopic cholecystectomy (Del Vecchio Blanco et al., 2021). Besides, patients who require surgical treatment due to hilar biliary stricture caused by stones can also be observed at present. However, surgical treatment methods for biliary deficit lesions crediting to iatrogenic bile duct injury, BA and hilar biliary stricture have many limitations and disadvantages. Therefore, only biliary-enteric anastomosis can be performed in most patients. Nevertheless, the biliary-enteric anastomosis reconstructs the digestive tract, and the anastomosis fails to function as the Oddis sphincter, which results in a series of postoperative complications such as reflux cholangitis, anastomotic leakage, anastomotic stricture, stone formation, biliary cirrhosis, and even carcinogenesis (Sampaziotis et al., 2017; Matthews et al., 1993; Tocchi et al., 2001; Laukkarinen et al., 2007; Kadaba et al., 2017). As a result, it is still a hot issue to preserve the function of the Oddis sphincter and make the reconstructed bile duct in line with the anatomical structure and physiological function of the normal bile duct. The appearance of organoids provides a certain possibility to preserve the function of the Oddis sphincter. Sampaziotis et al. pioneered the use of human bile duct epithelial organoids to repair the gallbladder and bile duct of mice (Sampaziotis et al., 2017; Tysoe et al., 2019). Another investigation conducted by Sampaziotis’ team suggest that organoids have the potential to be utilized for the restoration of human bile ducts (Sampaziotis et al., 2021). Similar findings were described in the study by Roos et al. (2021). These studies provide novel ideas and theoretical bases for the development of treatments for biliary defect diseases.

#### 2.1.5 Primary sclerosing cholangitis

Primary sclerosing cholangitis (PSC) is a cholestatic liver disease of unknown etiology that may be associated with autoimmunity, characterized by biliary inflammation and fibrosis (Reich et al., 2021). Eventually, cholestatic jaundice, cirrhosis, and liver failure develop as the disease progresses. Meanwhile, PSC is also one of the high-risk factors for developing bile duct cancer (Cordes et al., 2019; Dhillon et al., 2019). Soroka et al. collected bile samples from PSC patients using endoscopic retrograde cholangiography and then cultured organoids (Soroka et al., 2019). RNA sequencing showed that PSC-derived organoids changed the expression of 39 genes compared to control organoids. The expression of immune genes (such as HLA-DMA and CCL20) was increased in PSC-derived organoids, and these genes have previously been confirmed to be involved in PSC (Jiang and Karlsen, 2017).

#### 2.1.6 Hepatobiliary cancer

Currently, primary liver cancer, carcinoma of the bile duct and gallbladder are common primary tumors of the hepatobiliary system. Liver tumors are the sixth most prevalent and second most fatal cancer, with increasing incidence in the world (Bray et al., 2018). The current treatment for primary liver cancer is dominated by radical resection, supplemented by arterial chemoembolization, ablation, and sorafenib chemotherapy (Petrowsky et al., 2020; Benson et al., 2021). However, the treatment effect and overall prognosis of liver cancer are poor due to high malignancy and a high recurrence rate after radical resection (Vogel et al., 2018). Cholangiocarcinoma is the second most common malignant tumor of the hepatobiliary system, originating from the bile duct epithelium (Sarcognato et al., 2021). When patients are diagnosed with cholangiocarcinoma, most have unresectable tumors and fail to undergo surgery because of the specific anatomical position, insidious clinical symptoms and early neurovascular invasion and lymph node metastasis (Banales et al., 2020; Zhu, 2015). Although some patients are lucky to be treated with surgery, the tumor is prone to recurrence after the operation and the 5-year survival rate is less than 20% (Kamsa-Ard et al., 2019; Strijker et al., 2019; Cambridge et al., 2021; Cai et al., 2016). Cisplatin plus gemcitabine is the first-line chemotherapy regimen for patients in an advanced stage (Valle et al., 2010; Eckel and Schmid, 2014). Despite the significant effect, the prognosis is still unable to be improved (Koch et al., 2020). Gallbladder cancer, deriving from the gallbladder or the cystic duct, has high malignancy and is prone to metastasis in the early stage. Furthermore, it is usually insensitive to radiotherapy and chemotherapy (Kakaei et al., 2015). Like liver cancer and cholangiocarcinoma, most patients are in the advanced stage when the tumor is found (Schmidt et al., 2019). Therefore, it is considered one of the malignant tumors with poor prognoses in hepatobiliary surgery. Finding personalized and accurate therapy to enhance the outlook of individuals with liver and bile duct tumors continues to be a challenging issue in the medical field. As novel cancer models, the advent of organoids sheds light on this puzzle. At present, oncology research models mainly include human tumor cell lines, mouse models and human tumor xenograft mouse models. However, these models have some unavoidable shortcomings. For example, tumor cell lines lose the genetic heterogeneity of the original tissue during long-term subculture, which fails to reproduce the occurrence, development and metastasis of tumors. In the human tumor xenograft mouse model, it is unavoidable to use murine tumor stroma instead of human tumor stroma with low efficiency, long duration of tumor formation and high cost, limiting this model from being an excellent preclinical model (Bresnahan et al., 2020).

Tumoroids, organoids derived from cancer tissue, have distinct advantages in oncology research. Tumor-derived organoids, like non-tumor epithelial organoids, self-organize via cell-cell and cell-matrix interactions. It has been corroborated that there was a high degree of homology for gene expression profiles between primary carcinoma and liver tumoroids, especially in the expression of hepatocellular carcinoma markers (AFP, GPC3), hepatocyte markers (ALB, TTR, APOA1, APOE), bile duct epithelial markers (EpCAM, KRT19, S100A11) (Broutier et al., 2017). Several studies have demonstrated that biliary tract tumoroids robustly express bile duct epithelial markers (CK19, CK7, EpCAM, S100A6) (Wang et al., 2021; Saito et al., 2019; Maier et al., 2021).

Broutier’s team described a novel, near-physiological organoid culture system and extend the 3D culture system to the propagation of primary liver cancer organoids including HCC, cholangiocarcinoma (CC), and combined HCC/CC (Broutier et al., 2017; Broutier et al., 2016). In Nuciforo et al.’ study, poorly differentiated hepatic tumors organoids model can also be established derived from needle biopsies (Nuciforo et al., 2018). In 2019, organoids for biliary tract cancer (BTC) were developed from excised tumor tissues (Saito et al., 2019). Similarly, organoids for childhood liver cancers, such as hepatoblastoma (HB), have been developed using a 3D system (Saltsman et al., 2020). The use of surgical specimens from human or murine hepatomas has increasingly become the predominant method for creating liver cancer organoids. While the successful establishment rate of about 30% (Nuciforo et al., 2018), significantly lower than the reported success rates for establishing organoids of pancreatic and colorectal cancer. Thus far, multiple tumoroids have been developed to recapitulate HCC, CC, hepatoblastoma, BTC and combined HCC/CC, which have substantially contributed to liver and bile ducts cancer research for oncologists (Ren et al., 2023). Tumor-derived organoids replicate the histological structure, genomic landscape, gene expression, and tumorigenic potential of the original tumor, offering a novel *in vitro* model for cancer research. Tumoroids preserve the tumor’s original diversity and histopathological features both *in vitro* and after xenografting *in vivo*.

The integration of CRISPR/Cas9 and organoid technologies has greatly enhanced the development of tumor models, improving both tumor representation and the accuracy of gene effect predictions. Clevers et al. created human Primary liver cancer (PLC) tumoroids from healthy iPSCs by employing CRISPR-Cas9 to modify the BAP1 gene and furthermore, developed innovative PLC tumoroids by employing CRISPR-Cas9 technology to mutate four genes: NF1, SMAD4, PTEN, and TP53 (Artegiani et al., 2019). CRISPR-Cas9 technology is applicable in liver organoid development due to its genome-modulating capabilities (Artegiani et al., 2019). Artegiani et al. utilized CRISPR-Cas9-mediated homology-independent organoid transgenesis (CRISPR-HOT) technology to tag specific genes and sequences in human organoids (Artegiani et al., 2020). This technology facilitates organoid research by using fluorescent reporter genes to label and visualize specific molecules. This technology can also induce genetic changes to enhance the development of liver organoids from human fetal cells (Hendriks et al., 2021). CRISPR-HOT technology enables monitoring of cell fate, development, and division, as well as inducing genetic modifications in liver organoids.

Thus, the organoid is a good model to investigate the mechanisms of tumorigenesis, progression, metastasis and recurrence of hepatobiliary cancer. It is also an important tool to predict mutations and develop targets for targeted therapy. Moreover, organoids have the potential to be tools for marker discovery (Broutier et al., 2017). Tumoroids are widely utilized in anti-tumor drug screening as well as precision medicine, and this part is further discussed in the following section. The hepatobiliary organoids application as disease modeling examples are listed in Table 1.

**TABLE 1:** The examples of hepatobiliary organoids application as disease modeling are listed in the table.

Diseases		Species	Cell source	Expansion medium	Main findings	References
Hereditary disease	Alpha-1 antitrypsin deficiency (AATD)	Human	Bile duct cells	AdDMEM/F12, N2, B27, N-Acetylcysteine, gastrin, EGF, Rspo1, FGF10, HGF, Nicotinamide, A83.01, FSK.	Organoids from A1AT-deficiency patients can be expanded *in vitro* and mimic the *in vivo* pathology	Huch et al. (2015)
		Human	Bile duct cells	AdDMEM/F12, penicillin/streptomycin, Glutamax, Hepes, N-acetylcysteine, Rspo1, nicotinamide, gastrin Ⅰ, EGF, FGF, HGF, Rho kinase	Liver organoid model recapitulates the key features of Z-AAT deficiency including intracellular aggregation and lower secretion of AAT protein, and lower expression of ALB and APOB.	Gómez-Mariano et al. (2020)
	Alagille syndrome (ALGS)	Human	Bile duct cells	AdDMEM/F12, N2, B27, N-Acetylcysteine, gastrin, EGF, Rspo1, FGF10, HGF, Nicotinamide, A83.01, FSK.	Organoids from an ALGS patient reproduce the structural duct defects present in the biliary tree of these patients	Huch et al. (2015)
		Mice	Bile duct cells	AdDMEM/F12, N2, B27, N-Acetylcysteine, gastrin, EGF, Rspo1, FGF11, HGF, Nicotinamide	Establishment of bile duct–derived organoids from Jag1^Ndr/Ndr^ mice	Andersson et al. (2018)
		Human	iPSCs	RPMI, B27, LDN-193189, CHIR99021, A83-01, EGF, FGF10, HGF.	iPSC-hepatic organoids recapitulate the impaired bile duct formation that is characteristic of ALGS liver pathology, with reduced ability to form bile ducts and impaired regenerative ability	Guan et al. (2017)
	Cystic fibrosis (CF)	Human	iPSCs	William’s E medium, nicotinamide, sodium bicarbonate, 2-Phospho-L-ascorbic acid trisodium salt, sodium pyruvate, glucose, Hepes, ITS + premix, dexamethasone, Glutamax, penicillin, streptomycin, EGF.	iPSCs-cholangiocyte-like cells of CF patients model *in vitro* key features of CF-associated cholangiopathy; VX809 rescues the disease phenotype of CF cholangiopathy *in vitro*	Sampaziotis et al. (2015)
		Human	Bile duct cells	AdDMEM/F12, N2, B27, N-Acetylcystein, gastrin, EGF, FGF10, HGF, nicotinamide, A83.01, forskolin, Y27632, R-spondin, Noggin, Wnt, hES cell cloning recovery solution	ECO have cholangiocyte fate differentiation capacity but no potential for hepatocyte-like fate differentiation. ECO derived from a cystic fibrosis patient showed no CFTR channel activity	Verstegen et al. (2020)
	Wilson’s disease	Dog	Bile duct cells	AdvDMEM/F12, B27, N2, N-acetylcysteine, gastrin, EGF, R-spondin-1, nicotinamide, HGF, Noggin, Wnt3a, Y-27632, A83-01	Establishment of a long-term canine hepatic organoid culture. Successful gene supplementation in hepatic organoids of COMMD1-deficient dogs restores function	Nantasanti et al. (2015)
			Liver stem cells		The COMMD1-deficient organoid, after restoration of COMMD1 expression, were safely delivered as repeated autologous transplantations via the portal vein	Kruitwagen et al. (2020)
	Wolman disease	Human	iPSCs	AdvDMEM/F12, N2, retinoic acid (RA)/Hepatocyte Culture Medium (HCM), HGF, Dexamethasone, Oncostatin M	Multi-cellular human liver organoids of Wolman disease recapitulated key features of steatohepatitis, and organoid stiffening reflects the fibrosis severity. Severe steatohepatitis was rescued by FXR agonism-mediated reactive oxygen species suppression	Ouchi et al. (2019)
Viral hepatitis		Human	iPSCs, HUVECs, BM-MSCs	DMEM/12, GlutaMAX, HEPES, insulin	HBV infection in iPSC-liver organoids could recapitulate virus life cycle and virus induced hepatic dysfunction	Nie et al. (2018)
		Human	Hepatocytes	Ad+++, B27, N2, N-acetyl-L-cysteine, Rspo-1, Wnt3a, nicotinamide, recombinant human gastrin I, EGF, FGF10, HGF, forskolin, A83-01, Noggin, Y27632	Primary *ex vivo* HBV-infection model derived from healthy donor liver organoids after challenge with recombinant virus or HBV-infected patient serum	De Crignis et al. (2021)
Fatty liver		Human	iPSCs	William’s E medium, Dexamethasone, SBSB431542, DAPT, OSM.	End-stage hepatocytes derived from non-alcoholic steatohepatitis donors demonstrated spontaneous lipidosis without fatty acid supplementation, recapitulating a feature of NASH hepatocytes *in vivo*	Gurevich et al. (2020)
		Human	Liver stem cells	AdDMEM/F12, Pen/Strep, glutamax, Hepes, B27, NAC, Nicotinamide, R-spondin, N2, FGF-10, HGF, EGF, Gastrin, Forskolin, A83-01, Y27632	Expansion of primary liver stem cells/bipotent ductal organoids derived directly from irreversibly damaged non-alcoholic steatohepatitis patient liver, showing significant upregulation of liver fibrosis and tumor markers, and reduced passaging/growth capacity	McCarron et al. (2021)
Biliary atresia		Mice	Cholangiocytes	Mouse HepatiCult organoid growth medium supplemented with penicillin–streptomycin	First description of cholangiocyte injury in the organoids derived from intrahepatic bile ducts. Fibrogenic response of injured organoids was associated with increased cholangiocyte apoptosis and decreased cholangiocyte proliferation	Chusilp et al. (2020)
		Human	Cholangiocytes	AdDMEM/F12, penicillin/streptomycin, Glutamax, Hepes, B27, N2, N-acetylcysteine, RSPO1, Nicotinamide, Gastrin, EGF, FGF10, HGF, Forskolin, A83-01	Establishment of biliary organoids from liver biopsies of infants with biliary atresia. EGF + FGF2 treatment induced developmental markers, improved cell-cell junction and decreased epithelial permeability	Amarachintha et al. (2022)
Bile duct regeneration		Human	Cholangiocytes	William’s E medium, nicotinamide, sodium bicarbonate, 2-phospho-L-ascorbic acid trisodium salt, sodium pyruvate, glucose, HEPES, ITS + premix, dexamethasone, Glutamax, penicillin and streptomycin, EGF, R-spondin and DKK-1	Extrahepatic cholangiocyte organoids can self-organize into bile duct–like tubes after transplantation and can reconstruct the gallbladder wall and repair the biliary epithelium following transplantation into a mouse model of injury	Sampaziotis et al. (2017), Tysoe et al. (2019)
		Human	Cholangiocytes	AdvDMEM/F12, 1M HEPES, L-Ultraglutamine, Primocin, penicillin, streptomycine, N2, B27, N-Acetylcystein, RSPO1, Nicotinamide, Gastrin, EGF, FGF10, HGF, A83-01, Forskolin	Bile-cholangiocyte organoids originate from extrahepatic biliary tissue and are capable of repopulating human extrahepatic bile duct scaffolds. The cells obtain a transcriptomic profile more closely resembling primary cholangiocytes upon repopulation of scaffolds *in vitro*	Roos et al. (2021)
Primary sclerosing cholangitis		Human	Cholangiocytes	complete ADF medium, R-spondin, B27, nicotinamide, N-acetyl cysteine, N2, EGF, HGF, FGF10, gastrin, A83-01, forskolin	Bile-derived organoids retain features of cholangiopathies, including the ability to react to inflammatory stimuli by secreting chemokines and propagating immune-reactive phenotype	Soroka et al. (2019)
Hepatobiliary tumor	Hepatobiliary tumor	Human	Tumor cells	AdvDMEM/F12, penicillin/streptomycin, GlutaMAX‐I, HEPES, Primocin, B27, N‐acetyl‐l‐cysteine, EGF, FGF10, FGF‐basic, HGF, forskolin, A8301, Y27632, Rspo‐1, Wnt3a, Noggin	This study delineates heterogeneity of hepatobiliary tumor organoids and proposes that the collaboration of intra-tumoral heterogenic subpopulations renders malignant phenotypes and drug resistance	Zhao et al. (2021)
	Primary liver cancer	Human	Tumor cells	AdvDMEM/F12, Penicillin, Streptomycin, Glutamax, HEPES, B27, N2, N-Acetyl-L-cysteine, Rspo-1, nicotinamide, [Leu15]-Gastrin I, EGF, FGF10, HGF, Forskolin and A83-01	The tumorogenic potential, histological features and metastatic properties of primary liver cancer-derived derived organoids are preserved *in vivo*. Patient-derived organoids are powerful research tool for the drug screening	Broutier et al. (2017), Nuciforo et al. (2018), Saito et al. (2019), Saltsman et al. (2020)
	Extrahepatic cholangiocarcinoma and Gallbladder carcinoma	Human	Tumor cells	AdvDMEM/F12, Penicillin, Streptomycin, Glutamax, HEPES, B27, N2, gastrin, A83-01, Y-27632, EGF, FGF10, R-Spondin1, Noggin, Afamin/Wnt3a CM.	Biliary tract cancer patient-derived organoids show similar histological and genetic characteristics to the corresponding primary tumor tissues. Patient-derived organoids are powerful research tool for the drug screening	Wang et al. (2021), Saito et al. (2019), Ren et al. (2023)

### 2.2 Biobank

The establishment of organoid biobanks has been facilitated by advancements in the long-term preservation, storage, culturing, and expansion of organoids (Xie et al., 2023). Biobanks facilitate the standardized preservation and collection of PLC tumor samples along with their clinical data. As is mentioned in the previous sections, Broutier et al. established a biobank of PLC tumoroids from seven patients, maintaining the characteristics and expression profiles of the original tumors, including mutations in ARID2, ARID1A, TP53, KRAS, CTNNB1, and WNT1 (Broutier et al., 2017). And Nuciforo and colleagues created an HCC tumoroid biobank that replicated the histopathological and genetic characteristics of original tumors from 38 patients with poorly-differentiated tumors (Nuciforo et al., 2018). Xenograft models demonstrate that PLC tumoroid transplantation in experimental animals induces metastatic traits akin to the original tumors. These data suggest that the tumoroids biobank is suitable for disease modeling, drug testing and validation in PLC and hepatobiliary tumors. Ji’ team established a biobank of 65 patient-derived liver cancer organoids, encompassing 44 HCC organoids, 12 intrahepatic cholangiocarcinoma (ICC) organoids, and 4 combined HCC/CC organoids. These organoids comprehensively represent the histological and molecular characteristics of diverse liver cancer types, as determined by multiomics profiling, including genomic, epigenomic, transcriptomic, and proteomic analyses (Ji et al., 2023). Yang et al. established a PLC biobank was with 399 tumor organoids from 144 patients, accurately reflecting the histopathology and genomic characteristics of the original tumors. This biobank is effective for drug sensitivity screening, as demonstrated by *in vivo* models and patient responses (Yang et al., 2024).

### 2.3 Drug research and precision medicine

Precision medicine aims to enhance disease characterization at the molecular and genomic levels, thereby improving drug screening. Drug screening refers to the screening of new drugs or lead compounds with bioactivity from natural products or synthetic compounds. Due to the advantages described above, lots of studies have utilized organoids as ideal models for drug screening. Patient-specific tumoroids can be established in a short time by culturing tumor specimens obtained by biopsy or surgical resection from the patient (Eckel and Schmid, 2014). The drug screening platform based on patient-specific tumoroids tends to test the sensitivity of the tumor to anticancer drugs in a very short time, providing data support and guidance for individualized treatment.

Broutier et al. tested the sensitivity to 29 anticancer drugs in tumoroids originating from HCC, CC and combined HCC/CC and the results showed that except for CC-2 tumoroid was resistant to all anticancer drugs, and other tumoroids had their respective sensitive drugs (Broutier et al., 2017). Another study used diethylnitrosamine (DEN) to induce liver cancer in mice and generated liver tumoroids from these mice (Cao et al., 2019). Then, they performed drug sensitivity testing and found that 3 samples were sensitive to both sorafenib and regorafenib, 6 were sensitive to only sorafenib, and 4 were not sensitive to both sorafenib and regorafenib. Tissues from different regions of surgical cholangiocarcinoma specimens were obtained to generate 27 tumoroids, which were used to perform drug screening with 129 antitumor drugs (Li et al., 2019). The study showed that during the 129 antitumor drugs, most drugs were only effective against a few tumoroids. But bortezomib, romidepsin, prukamycin, idarubicin, panobinostat, carfilzomibhe and ixazomib were effective against all tumoroids and had moderate or higher killing activity against most tumoroids. Wang et al. constructed 5 gallbladder patient-derived tumoroids (GBC 1–5) and an extrahepatic cholangiocarcinoma (eCCA) patient-derived tumoroid, and found that GBC1 was sensitive to 5-fluorouracil, GBC2 was sensitive to gemcitabine and paclitaxel, GBC3 was sensitive to gemcitabine, GBC4 was sensitive to infigratinib and cisplatin, GBC4 was sensitive to paclitaxel and eCCA was sensitive to gemcitabine (Wang et al., 2021). Additionally, they found that treatment with 10 or 50 μM paclitaxel greatly decreased the growth rate of GBC5 tumoroid, indicating that organoids can be used to identify optimal drug doses. Similarly, a recent study cultured 3 bile duct tumoroids using patient-derived cholangiocarcinoma tissues and transplanted the tumoroids into immunodeficient NSG mice (Maier et al., 2021). Then, the mice formatting tumor successfully were utilized for further *in vivo* drug testing. The experimental results exhibited that tumors in mice treated with gemcitabine stopped growing, while tumors in control mice continued to grow and the response of mice treated with gemcitabine resembled human cholangiocarcinoma patients. Yuan et al. tested 20 targeted drugs approved by the FDA (Food and Drug Administration) that have minimal toxicity to normal gallbladder organoids. The findings indicate that histone deacetylase (HDAC) inhibitors can effectively reduce the growth of gallbladder tumoroids (Yuan et al., 2022).

Consequently, the conclusion can be drawn that different patients have different sensitivities to different chemotherapies or anticancer drugs. The establishment of patient-specific organoid models is able to provide a possibility for drug screening and evaluation of drug efficacy. Meanwhile, organoid xenografts originating from patients exhibited treatment responses analogous to the corresponding patient malignancies, which provide a direct and reliable basis to guide the medication regimen. The organoid is a precision medicine-oriented and efficient preclinical model and has value as an alternative to *in vivo* models.

Other applications of hepatobiliary organoids for medicine research also include drug resistance and toxicity assessment (Zhao et al., 2021; Leung et al., 2020). In addition, hepatobiliary organoids can also be used as a good *in vitro* prediction model of drug hepatotoxicity. In recent years, some research teams have seen the potential of liver organoids and applied them to the assessment of drug metabolic parameters and toxicity, which have been developed to study and predict drug-induced liver injury (Brooks et al., 2021).

While patient-derived organoids (PDOs) are gaining traction in therapeutic screening, various challenges need to be overcome to unlock their full potential. Firstly, the successful establishment of organoids relies on the availability of fresh and viable tissue samples. However, acquiring adequate and high-quality tissue samples for organoid culture is challenging, particularly for some specific tumor types. A further challenge involves the scarcity of patient-derived samples and the ethical issues related to their acquisition. Alternative sources such as minimally invasive procedures or liquid biopsies may be the direction of exploration.

Another major challenge is from the absence of standardized methods for generating and culturing PDOs. Standard methods are essential to ensure the reliability and reproducibility of PDOs as a therapeutic screening model. Differing protocols used by laboratories for their isolation, expansion, and differentiation may lead to the variability in organoid quality and characteristics, which can hinder the comparison of results across studies and the reproducibility of findings in various laboratories.

Organoids should deliver swift outcomes to inform treatment choices within a clinically relevant period. For postoperative adjuvant chemotherapy, 1–3 weeks may be acceptable interval for a drug sensitivity test. However, for neoadjuvant chemotherapy or those advanced tumors, drug screening tests are needed as soon as possible. Efforts are essential to streamline the workflow and minimize the turnaround time for organoid generation and drug sensitivity testing.

Organoids may become contaminated with normal cells during the culturing process. Implementing quality control measures for organoids is crucial prior to drug sensitivity testing. For example, Next-generation sequencing (NGS) is conducted before drug sensitivity testing to verify the presence of key mutations in organoids that influence drug response.

Finally, correlating organoid drug sensitivity testing results with clinical outcomes is crucial to validate its effectiveness in guiding treatment decisions and enhancing patient outcomes. Extensive longitudinal studies involving larger patient cohorts are essential to assess the clinical efficacy and performance of drug sensitivity testing.

### 2.4 Regenerative medicine

Currently, only liver transplantation can treat various end-stage liver diseases, but the shortage of donors is always a difficulty. Moreover, as previously described, some patients suffer from bile duct defects due to congenital or acquired causes and there is currently no effective treatment. To overcome this dilemma, an increasing number of researchers valued the value of organoids in regenerative medicine. Yang’ team constructed 3D bio-printed hepato-organoids by 3D printing technology and transplanted them into immunodeficient mice with tyrosinemia type Ⅰ and liver failure (Yang et al., 2021). After being transplanted, the organoids have the ability to develop functional vascular system. Furthermore, the previous section on disease modeling on “Biliary atresia and bile duct defects” mentioned the utilization of human bile duct organoids to restore the gallbladder, bile ducts, and intrahepatic bile ducts in isolated human livers through various studies (Sampaziotis et al., 2017; Tysoe et al., 2019; Sampaziotis et al., 2021).

Autologous organoids may not be feasible for all regenerative medicine applications due to various limitations. Organoid derivation is a time-consuming process, making it unsuitable for patients with acute liver failure who need immediate off-the-shelf regenerative medicine solutions. Second, patient-derived autologous primary organoids might still be influenced by the disease, leading to diminished organ regeneration capacity. Furthermore, access to primary tissue may be unattainable in certain cases, such as cholangiocytes in vanishing bile duct syndrome.

## 3 Limitations and future perspectives

The past decade has witnessed dramatic progress in organoid technology. Organoids possess distinct advantages as they replicate almost physiological circumstances and maintain parental genetic stability. Disease modeling and drug screening studies can utilize these cells or tissues, which can also be used to treat disorders caused by mutations by reversing the disease-causing mutation. Moreover, organoids exhibit rapid growth and a high rate of success in culture, potentially addressing the issue of low efficiency in forming tumors in patient-derived tumor xenograft models. Nevertheless, the current state of the technology is not fully developed, and numerous obstacles remain that must be surmounted.

Lack of microenvironment sometimes, especially in ASC-derived organoids is the first limitation. Organoid technology serves as an intermediary between cell lines and *in vivo* models, yet it often lacks critical components such as stromal, immune, and vascular endothelial cells needed for thorough modeling. For instance, liver organoids frequently miss hepatocyte zonation and key elements involved in the pathogenesis of metabolic fatty liver disease, including vasculature, immune cells, and neural innervation. This impedes their capability to precisely predict clinical outcomes and prognoses.

As is written in the drug screening section, globally standardized protocols for organoid establishment and quality control are urgently needed. The organoid industry faces challenges due to insufficient standardization, a problem intensified by the swift advancements in engineered organoids. Reproducibility is influenced by batch variations such as patient tissue heterogeneity and the timing and method of iPSC induction, as well as culture conditions like cytokine concentration, matrix gel concentration and composition, and the composition and structure of cells and organoids. Addressing these challenges necessitates collaboration among biomedical scientists, clinicians, and regulatory bodies to standardize organoid technology, thereby easing its transition from research to clinical applications and enabling large-scale organoid production for drug screening.

Relatively higher expenditure compared to traditional models is equally noteworthy. Organoid establishment, maintenance, and passages are costly. The high price of growth factors and medium additives restrict the popularization of organoid culture technology. Only a few laboratories are able to perform organoid culture. To some extent, economic pressures have limited the widespread adoption of organoid technology. Another disadvantage of organoid culture is that it is time consuming, which has also been discussed in the chapter of drug screening.

Furthermore, tissue samples prepared for organoid generation are only small parts of the whole tumor. The higher heterogeneity of tumors questions the reliability of substituting small pieces for whole tumor tissues. Tissue extraction from different sites of the same tumors might better reflect tumor heterogeneity and reliably facilitate cancer translational research.

Organoid technology currently struggles to replicate the complexity of patient-specific immune environments. While coculturing tumoroids with immune cells enhances the modeling of tumor-immune interactions and treatment effects, certain challenges may impede precise modeling and prediction of immunotherapy responses. Different tumor types exhibit unique immune components and varying cell quantities, influencing the immune cell composition in early tumoroid culture and the potential for maintaining and expanding these immune cells. Tumors vary in immune cell composition, with some containing diverse and complex immune cells, while others have immune cells only in the surrounding stroma or lack them entirely. In addition, although preserved immune cells can be maintained initially, they may be lost and diluted over time. Inaccurate modeling of the tumor immune environment limits the utility of organoids in translational and precision medicine.

Vascularization of organoids is still a major challenge. Although implantation of organoids into animals or coculture systems promotes organoid vascularization, these methods only endow organoids with vascular characteristics but not functional perfusion vessels (Shirure et al., 2021). The current microfluidic platform used to establish vascularized organoids is crude and semi-adjustable, and it is affected by multiple factors, including the concentration and composition of cytokines and flow rate. More accurate and flexibly controllable and detectable microfluidic platforms are urgently needed for better vascularization of organoids and accurate prediction of responses to antiangiogenic therapies.

The past decade has witnessed dramatic progress in organoid technology. Organoids faithfully maintain the histological and gene expression characteristics of native tissue, making it the important preclinical models for studying diseases and developing therapies. The use of hepatobiliary organoids technology presents a unique opportunity to investigate the pathophysiological process and disorders of the human hepatobiliary system. These innovative preclinical models hold great potential for future applications. Nevertheless, the organoid method is currently in its early phase and possesses certain limitations. Vascularization of organoids remains a hotspot in tissue engineering. In the future, how to combine new technologies (CRISPR-HOT, Organ-on-a-chip and so on) with organoid and accelerate translational applications is important.
